# Genome-Wide InDel Marker Development and Genetic Diversity Analysis of 52 Tomato Germplasm Accessions

**DOI:** 10.3390/plants15071118

**Published:** 2026-04-06

**Authors:** Chenjiao Huang, Di Ge, Yaxuan Zhang, Zhiye Ge, Yicheng Wu, Qianrong Zhang, Yunxia Zhao, Chonghui Ji

**Affiliations:** 1 Center for Genomics and Biotechnology, Fujian Provincial Key Laboratory of Haixia Plant Systems Biology, Haixia Institute of Science and Technology, College of Life Sciences, Fujian Agriculture and Forestry University, Fuzhou 350002, China; huangchenjiao01@163.com (C.H.); gedi0000@163.com (D.G.); zyxxuan2022@163.com (Y.Z.); akikulihouya@163.com (Z.G.); wuyicheng2759@163.com (Y.W.); 2 Fujian Key Laboratory of Vegetable Genetics and Breeding, Crops Research Institute, Fujian Academy of Agricultural Sciences, Fuzhou 350002, China; zhangqianrong@faas.cn; 3 Institute of Horticulture, Ningxia Academy of Agriculture and Forestry Sciences, Yinchuan 750002, China

**Keywords:** tomato germplasm, molecular markers, InDel makers, genetic diversity

## Abstract

To address the challenges of narrow genetic backgrounds and low phenotypic selection efficiency in tomato breeding, comparative genomics was applied. Based on the genomic sequences of five tomato varieties (‘Micro-Tom’, ‘Moneymaker’, ‘M82’, ‘Heinz 1706’, and ‘LA2093’), a total of 285,796 InDel loci were preliminarily identified. Based on these loci, a total of 255 pairs of molecular markers were developed. Subsequently, based on InDel length, polymorphism, and electrophoretic performance, 63 InDel markers with stable amplification, clear polymorphic bands, and coverage across all 12 chromosomes were rigorously selected. These markers were subsequently used to analyze the genetic diversity of 52 tomato germplasm resources. The polymorphism information content (PIC) values of the markers ranged from 0.074 to 0.402, with an average of 0.2804. Cluster analysis based on InDel genotyping data divided the 52 germplasm samples into four distinct groups with significant genetic differentiation, which was validated in conjunction with previously collected phenotypic data from the 52 tomato germplasm resources. Furthermore, a set of core InDel primer combinations (24 pairs) was selected to construct unique DNA fingerprint profiles for each germplasm group. Overall, the InDel markers developed in this study provide an efficient tool for evaluating genetic diversity in tomato germplasm and offer a reliable molecular basis for germplasm identification, heterosis prediction, and marker-assisted breeding, thereby facilitating the development of improved tomato cultivars.

## 1. Introduction

Tomato (*Solanum lycopersicum*) is a nutritious, brightly colored vegetable crop with a distinctive aroma. Its high content of lycopene, vitamin C, beta-carotene, and other bioactive compounds, together with its sweet and sour flavor, makes it an indispensable part of the daily diet, and gives it considerable economic and nutritional value [1]. Tomatoes originated in the Andes Mountains of South America. After being introduced to Europe in the 15th century, and through hundreds of years of domestication and improvement, the morphological characteristics and agronomic traits of tomatoes have been significantly enhanced, gradually developing into the modern cultivated tomato [2]. During the long-term domestication and breeding of tomatoes, continuous artificial selection and genetic bottleneck effects have significantly reduced the genetic diversity of cultivated varieties, posing a major challenge to the genetic improvement of important agronomic traits [3].

In recent years, with the rapid development of molecular breeding technology, molecular marker-assisted selection of genes for superior agronomic traits has gradually become an important strategy of tomato breeding [4]. Molecular breeding mainly involves developing molecular markers that are closely linked to target genes, followed by the use of these markers to screen for desired traits during the breeding process [5,6,7]. Molecular markers are genetic markers based on nucleotide sequence variation among individuals. They reflect genetic polymorphism at the DNA level and are widely used in germplasm identification, marker-assisted breeding, and genetic improvement. Compared to traditional morphological, cytological, and protein markers, molecular markers have several advantages. First, they are not affected or limited by environmental conditions or gene expression, making them more reliable and stable in genetic studies. Second, a large number of molecular markers are available across the genome, providing more comprehensive and detailed data for genetic research [8,9]. Based on the methods to detect DNA polymorphisms, molecular markers can generally be divided into four categories: (1) RFLP (Restriction Fragment Length Polymorphism) markers based on Southern hybridization detection; (2) PCR-based molecular markers, such as RAPD (Random Amplified Polymorphic DNA), SSRs (Simple Sequence Repeats), ISSRs (Inter-simple Sequence Repeats), and SRAPs (Sequence-related Amplified Polymorphisms) [10]; (3) markers based on a combination of PCR and restriction enzyme digestion, such as AFLPs (Amplified Fragment Length Polymorphisms) and CAPSs (Cleaved Amplified Polymorphic Sequences) [11]; and (4) newly developed markers based on sequence variation, such as InDels (Insertion-Deletions) and SNPs (Single Nucleotide Polymorphisms) [12,13]. These marker systems play an important role in genetic polymorphism studies, and provide strong technical support for germplasm identification, studies of genetic evolution, and molecular breeding [14,15]. Among numerous molecular markers, InDel markers are widely used due to their simple structure, high polymorphism rate, co-dominant characteristics, and close linkage with target traits. These markers have been successfully applied in tomato genetic improvement studies, covering key breeding objectives such as disease resistance, quality enhancement, and stress tolerance. InDel markers are widely distributed in the genome, easy to detect, and highly reproducible, making them very suitable for high-density molecular marker development and are widely applied in gene mapping, association studies, and genetic linkage map construction.

With the completion of the tomato reference genome sequencing and assembly [16,17,18], many important traits in tomato have been finely mapped. To date, more than 100 genes in tomatoes have been cloned or precisely mapped. Although considerable progress has been made in identifying functional genes controlling important tomato traits, there is still a lack of rapid, efficient, and low-cost molecular markers suitable for breeding applications [19,20]. Therefore, the development of tomato DNA molecular markers is expected to provide precise and efficient tools for marker-assisted breeding, molecular genetic mapping, and the study of functional genes involved in abiotic stress responses, thereby promoting rapid progress in related research fields [21,22].

In this study, genomic sequences from five tomato varieties—‘Micro-Tom’, ‘Moneymaker’, ‘M82’, ‘Heinz 1706’, and ‘LA2093’—were used to identify genome-wide InDel loci and develop a set of easily detectable molecular markers. A total of 63 InDel markers distributed across the 12 tomato chromosomes were selected and subsequently used to analyze the genetic diversity of 52 tomato germplasm accessions. In addition, DNA fingerprinting profiles were constructed based on these markers. The results provide useful molecular tools for germplasm identification, genetic diversity evaluation, and marker-assisted breeding, thereby contributing to the efficient utilization and genetic improvement of tomato germplasm resources.

## 2. Results

### 2.1. Identification and Statistical Analysis of Genome-Wide InDel Molecular Markers

**Figure 1:**
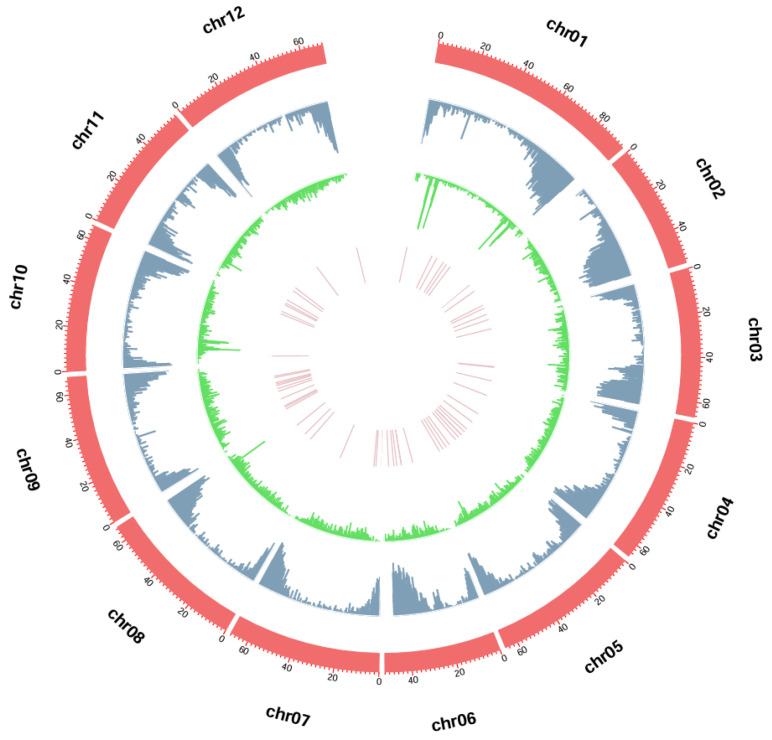
Genome-wide distribution of InDel loci and selected molecular markers in the tomato genome. The outermost layer represents chromosomes (red). From the second ring inwards, the layers are arranged in the order: genes (blue), InDels (≥6 bp, green), and selected InDel molecular markers (pink). Each 50 kb interval was used as a statistical unit.

To develop InDel molecular markers across the entire tomato genome, conserved sequence fragments containing InDel polymorphisms were identified based on genomic data from five tomato varieties: ‘Micro-Tom’, ‘Moneymaker’, ‘M82’, ‘Heinz 1706’, and ‘LA2093’. A total of 285,796 InDel loci were detected, including 121,933 insertions (42.66%) and 163,863 deletions (57.34%). To better illustrate the distribution of InDel molecular markers across the chromosomes, genes, InDel loci, and selected molecular markers were statistically analyzed in 50 kb intervals. As shown in Figure 1, the outermost circle indicates the chromosomal start positions, while genes (blue), InDel loci (green), and selected molecular markers (pink) are sequentially displayed from the outer to the inner tracks. The number of InDel loci across the 12 chromosomes ranged from 19,553 (Chr11) to 27,078 (Chr09) (Table 1). In terms of chromosomal distribution, the number of InDel sites ranged from 19,553 (Chr11) to 27,078 (Chr09), accounting for 6.84% to 9.47% of the total InDels, respectively. After correcting for chromosome length, there was no significant difference in InDel density among the chromosomes. However, as illustrated in Figure 1, the distribution of InDel sites along individual chromosomes was uneven. Regions with high gene densities contained fewer InDels, whereas InDels were preferentially enriched in gene-sparse regions. Furthermore, InDel density near centromeric regions was significantly reduced, consistent with the reduced detection efficiency in regions rich in repetitive sequences. The final set of 63 InDel markers (pink track) was evenly distributed across the genome, providing comprehensive coverage for subsequent genetic analyses.

### 2.2. Development and Screening of InDel Markers

**Figure 2:**
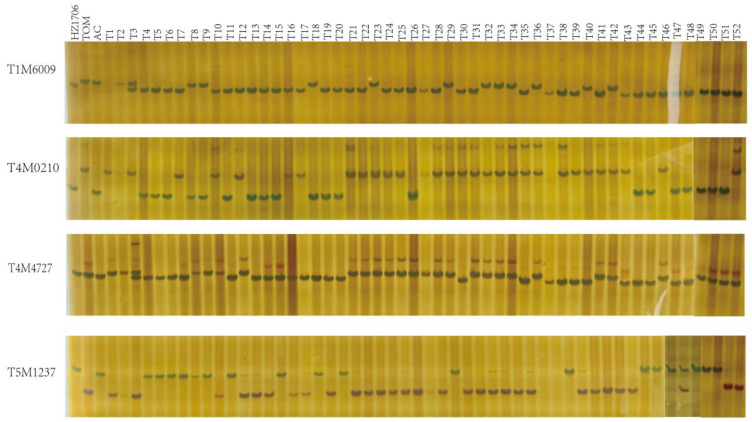
PCR amplification profiles of 52 tomato accessions using four InDel primer pairs. *HZ1706*, *TOM* and *AC* are the reference genome varieties, while T1–T52 indicate the 52 tomato accessions.

**Figure 3:**
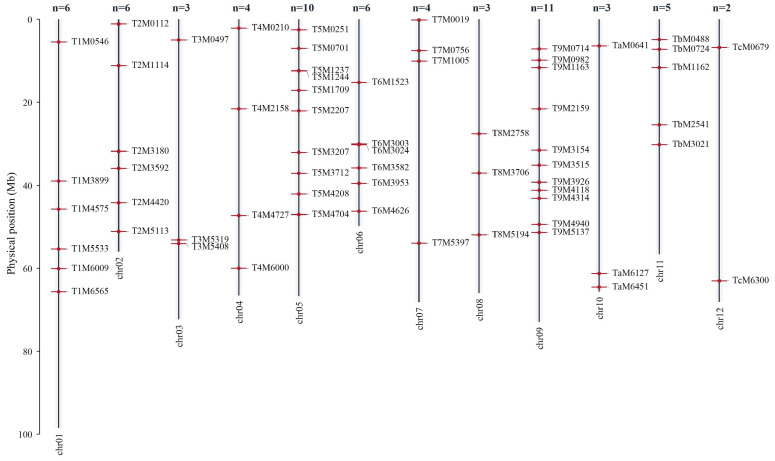
Chromosomal distribution of the 63 core InDel markers in the tomato genome.

To establish a high-quality set of InDel markers, loci with InDel lengths of ≥6 bp and polymorphisms between at least two tomato accessions were selected for marker development. A total of 255 candidate InDel loci distributed across the 12 tomato chromosomes were identified for further development of PCR-based markers (Table S1). Target sequences of 70–150 bp flanking the corresponding InDel loci were extracted as templates for primer design. PCR amplification was subsequently performed using DNA from 52 tomato germplasm accessions to evaluate the amplification efficiency and polymorphism of the designed primers. Based on the clarity, reproducibility, and polymorphism of the amplified bands, loci showing no or low polymorphism were discarded. Ultimately, 63 InDel markers (24.7%) that could be stably amplified and displayed clear polymorphism were selected. The PCR amplification results of 10 representative InDel primer pairs across the 52 tomato accessions are presented in Figure S1, while Figure 2 shows the amplification profiles of four representative InDel markers. Detailed information for the 63 InDel primer pairs is provided in Table S2. These markers are distributed across the entire tomato genome, with an average of 5.25 markers per chromosome. Among the 12 chromosomes, chromosome 12 contains the fewest markers (2), whereas chromosome 9 contains the most markers (11) (Figure 3).

### 2.3. Polymorphism Analysis of InDel Markers

Polymorphism Information Content (PIC) is an important indicator for measuring the polymorphism of genetic markers. A higher PIC value indicates greater marker polymorphism and provides more genetic information. We analyzed 52 tomato accessions using 63 core InDel markers. The results showed that the number of alleles amplified by each InDel marker ranged from 2 to 3, and the PIC values ranged from 0.038 to 0.402 (Table 2), with an average PIC of 0.307. Among these markers, 48 markers exhibited relatively high polymorphism (PIC > 0.25). These results demonstrate that the InDel markers developed in this study show moderate to high levels of polymorphism and are suitable for genetic diversity analysis of tomato germplasm.

### 2.4. Application of InDel Markers in Tomato Germplasm Analysis

**Figure 4:**
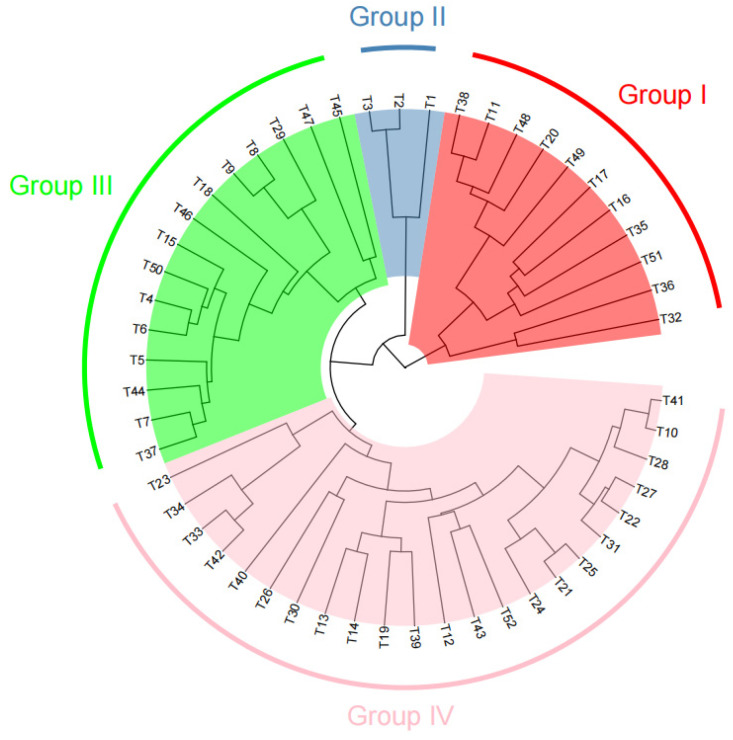
Cluster analysis of 52 tomato germplasm accessions based on InDel molecular markers.

A Neighbor-Joining (NJ) phylogenetic tree was constructed based on the genetic distance matrix to evaluate the genetic relationships among the 52 tomato accessions. The accessions were classified into four major groups (G1–G4) (Figure 4). Group G1 contained 11 accessions that shared similar phenotypic characteristics, including determinate growth habit, yellow or light-yellow flowers, red fruits, round or oblate fruit shape, and comparable soluble solid contents, suggesting a relatively narrow genetic background. Group G2 consisted of three accessions, all of which were wild small-fruited tomatoes. Group G3 included 15 accessions that were predominantly characterized by an indeterminate growth habit, red fruits with round or oblate shapes, and medium to high soluble solid contents. Group G4 represented the largest and most genetically diverse group, comprising 23 accessions, most of which were cherry tomato types. Overall, the developed InDel markers effectively distinguished different tomato germplasm types and revealed clear phylogenetic relationships among the tested accessions.

**Figure 5:**
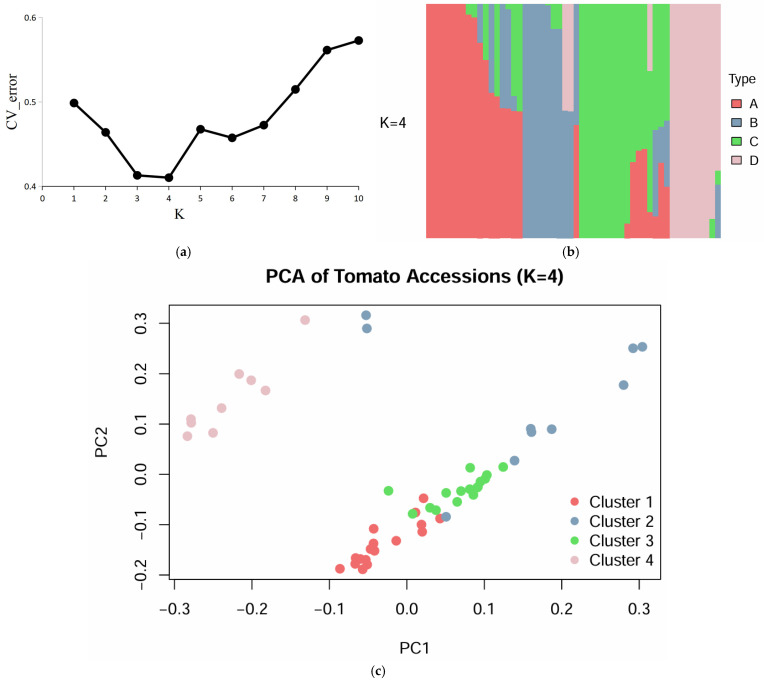
Population structure and principal component analysis of 52 tomato germplasm accessions. (**a**) The cross-validation error (CV_error) curve for determining the optimal number of genetic clusters; (**b**) Population structure bar plot of the 52 tomato accessions; (**c**) Principal Component Analysis (PCA) plot.

To further investigate the population structure, STRUCTURE analysis was performed. The cross-validation error (CV_error) curve indicated that the optimal number of genetic clusters was K = 4 (Figure 5a). The corresponding population structure plot (Figure 5b) showed that the 52 tomato accessions could be clearly divided into four genetic subpopulations (A, B, C, and D). Accessions within each subpopulation exhibited relatively homogeneous genetic composition, while clear genetic differentiation was observed among subpopulations, indicating distinct genetic backgrounds among the tested materials.

Principal component analysis (PCA) further supported the population structure results. In the PCA plot (Figure 5c), the first two principal components (PC1 and PC2) clearly separated the 52 accessions into four groups, which was consistent with the STRUCTURE analysis (K = 4). This indicates that the genetic differences among the four groups can be effectively distinguished through principal component analysis, further verifying the reliability of the population structure division. The different groups show obvious spatial distribution differences in the PCA plot, reflecting the independence of genetic variation among the groups, and also demonstrating that the molecular markers used have high polymorphism and discriminative power, fully revealing the genetic diversity among these tomato accessions.

### 2.5. Construction of DNA Fingerprinting

The phenotypic survey of the 52 tomato accessions revealed considerable morphological variation. Among them, 31 accessions (60%) exhibited an indeterminate growth habit, characterized by continuous flowering and fruiting with a relatively long growing season, whereas 21 accessions (40%) were determinate with a more concentrated growth cycle. Leaf morphology was classified into four major types: compound narrow leaves, compound broad leaves, common leaves, and sweet potato leaves. Among these, compound narrow-leaf types accounted for the highest proportion (approximately 45%), including accessions such as T20, T49, and T29. Inflorescence types were mainly dichotomous (approximately 60%) and simple (approximately 35%). Fruit shapes were predominantly round or oblate. The number of locules ranged from 1.88 to 9. Approximately 60% of the accessions had 2–4 locules, whereas about 40% contained 6–9 locules (Table S3). These results indicate substantial phenotypic diversity among the tested tomato germplasm.

All 63 InDel markers developed in this study exhibited clear polymorphism across the 52 tomato accessions, enabling complete discrimination among the tested varieties. Therefore, these markers can be used for variety identification and DNA fingerprint construction in tomato germplasm.

From the 12 tomato chromosomes, two to three biallelic InDel markers with relatively high PIC values were selected per chromosome to construct the DNA fingerprinting system. A total of 24 markers were selected, including T1M0546, T1M6565, T2M0112, T2M3180, T3M5319, T4M0210, T4M6000, T5M0701, T5M1237, T5M2207, T6M3024, T6M3582, T7M0019, T7M5397, T8M2758, T8M5194, T9M0714, T9M0982, TaM0641, TaM6451, TbM0724, TbM1162, TbM3021, and TcM0679. The genotyping results of these 24 markers across the 52 tomato accessions were recorded based on banding patterns. The absence of a band was denoted as “0”, while the presence of bands was represented by codes “1” to “4”. Specifically, “1” indicated a band consistent with the Heinz 1706 reference genome; “2” indicated the presence of an InDel relative to the Heinz 1706 genome; “3” represented heterozygous bands; and “4” indicated an InDel different in length from the type represented by code “2”. Based on these scoring criteria, the genotypes of the 24 markers were converted into 24-digit genotype codes to construct DNA fingerprints for the 52 tomato accessions (Table 3).

## 3. Discussion

Molecular markers have become essential tools for crop genetic research and modern breeding programs. With the rapid development of high-throughput sequencing technologies and the continuous reduction in sequencing costs, multiple reference genomes of tomato and its related wild species have been released in recent years [23,24,25]. These genomic resources have enabled the identification of large numbers of sequence variations across the tomato genome, thereby facilitating the development of highly efficient molecular markers.

Compared with early molecular marker systems such as RFLP and RAPD, genome-based markers offer higher resolution and reliability for genetic analysis. Among the currently available markers, SNPs represent the most abundant type of genetic variation in plant genomes; however, their detection often requires specialized equipment and relatively high costs, which limits their routine application in breeding programs. In contrast, SSR and InDel markers can be easily detected through PCR amplification followed by gel electrophoresis. Nevertheless, SSR loci consist of tandem repeat sequences, and large variations in repeat numbers may affect primer binding efficiency and amplification stability. InDel markers, by contrast, are widely distributed across the genome and are typically flanked by conserved sequences, which allows for the design of primers with higher specificity and more stable amplification efficiency [26].

In the present study, genome sequence data from five tomato accessions were compared to identify candidate insertion–deletion loci, from which, 63 core InDel markers with stable polymorphism were developed and evenly distributed across the 12 chromosomes of the tomato genome. The polymorphism and stability of these markers were validated using 52 tomato germplasm accessions. The results indicate that the developed InDel markers possess good amplification stability and relatively high polymorphism, making them suitable for genetic diversity analysis and germplasm identification. In addition, the wide application of InDel markers may facilitate more accurate analyses of population structure and pedigree relationships in tomato breeding materials, thereby contributing to studies on tomato genetic evolution and germplasm classification [27,28].

Germplasm resources represent the fundamental basis for crop genetic improvement. Evaluating the genetic diversity of tomato germplasm is essential for broadening the genetic base and identifying valuable breeding materials [29,30]. In this study, cluster analysis based on InDel markers grouped the 52 tomato accessions into four major clusters. The population structure analysis using STRUCTURE (V2.3.4) software produced results largely consistent with the clustering analysis, further supporting the reliability of the developed markers. Notably, three wild small-fruited tomato accessions (T1, T2, and T3) formed an independent subgroup, whereas the remaining accessions belonged to cultivated tomato groups. Furthermore, the clustering patterns were closely associated with several phenotypic traits, such as growth habit, fruit shape, and fruit color, indicating that the InDel markers developed in this study can effectively differentiate different types of tomato germplasm and reflect their phylogenetic relationships.

In this study, cluster analysis based on InDel markers can roughly divide 52 tomato germplasm accessions into four major clusters. At the same time, the population structure of the 52 tomato germplasm accessions was also analyzed using Structure software, and the population analysis results were basically consistent with the cluster analysis results. Three wild small-fruited tomato germplasm accessions, T1, T2, and T3, constituted a separate subgroup, while the remaining germplasm were cultivated tomatoes. The results of the cluster analysis showed a significant correlation with their traits (such as growth type, fruit shape, and fruit color), indicating that the developed InDel markers can effectively distinguish different types of tomato varieties or germplasm resources and reflect the phylogenetic relationships among the tested materials. These results demonstrate that the tomato germplasm analyzed in this study contains substantial genetic variation and provides valuable genetic resources for future breeding programs. The developed marker system may also support various breeding applications, including QTL mapping of important agronomic traits, parental selection, and germplasm identification. By enabling breeders to select parental materials with complementary genetic backgrounds according to breeding objectives—such as high yield, improved fruit quality, or enhanced storage and transport tolerance—the use of these markers may accelerate the process of tomato variety improvement.

Overall, the InDel markers developed in this study exhibit good polymorphism and genome-wide distribution across all 12 tomato chromosomes. These markers are easy to use and provide reliable molecular tools for tomato genetic diversity analysis, germplasm identification and evaluation, functional gene mining, marker-assisted selection, and variety purity testing.

## 4. Materials and Methods

### 4.1. Test Materials

This study selected five tomato germplasm accessions—cultivated varieties *S. lycopersicum* ‘Micro-Tom’, ‘Moneymaker’, ‘M82’, and ‘Heinz 1706’, and wild tomato *S. pimpinellifolium* ‘LA2093’—for InDel identification. In addition, 52 tomato resources (Table S3) were used for genetic diversity analysis. This batch of tomato germplasm resources includes various types such as wild small-fruited tomatoes, cherry tomatoes, and highly inbred self-pollinated lines. Among them, wild small-fruited tomatoes retain the original genetic characteristics of tomatoes; cherry tomatoes combine both edible value and genetic diversity; and highly inbred self-pollinated lines have a stable genetic background. These 52 varieties were provided by the Ningxia Academy of Agriculture and Forestry Sciences in Yinchuan, Ningxia Hui Autonomous Region, and their agronomic traits were observed and recorded.

### 4.2. Acquisition of the Reference Genome

The tomato reference genomes for ‘Micro-Tom’, ‘Moneymaker’, ‘M82’, ‘Heinz 1706’, and ‘LA2093’ were obtained from the National Center for Biotechnology Information (NCBI) database or the tomato database (http://solomics.agis.org.cn/). Download links for ‘Micro-Tom’: https://www.ncbi.nlm.nih.gov/pmc/articles/PMC11897730/ (accessed on 15 January 2025); ‘Moneymaker’: http://solomics.agis.org.cn/tomato/datasource/2/ (accessed on 15 January 2025); ‘M82’: https://www.ncbi.nlm.nih.gov/assembly/GCA_900008105.1/ (accessed on 15 January 2025); ‘Heinz 1706’: https://www.ncbi.nlm.nih.gov/datasets/genome/GCF_000188115.4/ (accessed on 15 January 2025); ‘LA2093’: https://www.ncbi.nlm.nih.gov/sra/?term=LA2093+Solanum+pimpinellifolium (accessed on 15 January 2025).

### 4.3. Indel Molecular Marker Screening and Primer Design

Based on Indel sites obtained from whole-genome alignment (a total of 285,796 sites), the following stepwise screening was conducted to obtain candidate sites suitable for subsequent marker development.

#### 4.3.1. Basic Filtering

The raw Indel set was initially filtered using BCF tools (v1.15), retaining sites that met the following criteria: Quality value (QUAL) ≥ 30; Indel length greater than or equal to 6 bp; Exclude Indels located in centromeres, telomeres, and known tandem repeat regions (based on Repeat Masker annotation); Extract 200 bp sequences on both sides of the Indel and align them with the reference genome to ensure they are located in non-repetitive, single-copy regions, avoiding amplification complexity caused by repeated elements.

#### 4.3.2. Genome Distribution Screening

To obtain a genome-wide evenly distributed marker set, Indels passing the basic filtering were sorted according to their physical chromosomal positions, and neighboring sites less than 100 kb apart were removed to ensure uniform marker distribution along the chromosomes. Additionally, based on genome annotation information, Indels were categorized into genic and intergenic regions, and sites were retained in proportion to balance the functional distribution of markers.

#### 4.3.3. Primer Design and Amplifiability Evaluation

For the retained Indel sites, 250 bp flanking sequences upstream and downstream were extracted, and InDel marker primers were designed using Primer 6.0 software. Primer design parameters were set as follows: primer length of 21–30 bp, Tm of 57–62 °C, GC content of 40–70%, product size of 70–150 bp, and primers should avoid forming dimers or hairpins with themselves or each other. Sites for which qualified primers could not be designed were excluded.

#### 4.3.4. Population Polymorphism and Experimental Validation

Four tomato materials with high genetic diversity were selected to perform PCR amplification and polyacrylamide gel electrophoresis testing on the above sites. Sites with failed amplification, blurry bands, or non-specific bands were excluded. Polyacrylamide gel electrophoresis was further used to confirm the length differences between Indels from the two parental materials, retaining sites with clear differences and single peak patterns.

#### 4.3.5. Final Marker Set

Through the above stepwise screening, 255 Indel molecular markers were ultimately obtained, which are evenly distributed on the genome, stably amplifiable, and highly polymorphic, suitable for subsequent genetic analysis and marker development. Among them, 63 markers showed significant population polymorphism in these 52 tomato varieties. The primers for the selected markers were synthesized by Shanghai Sangong Biotechnology Co., Ltd. (Shanghai, China), and were labeled using the naming format ‘Chromosome number + primer position.’

### 4.4. PCR Amplification and Polyacrylamide Gel Electrophoresis

PCR amplification and detection of the screened InDel marker were performed using 52 tomato materials. When the third true leaf of each of the 52 material samples was fully expanded, healthy seedlings were selected, and small amounts of young leaves were taken and placed in 2 mL centrifuge tubes. Genomic DNA was extracted using the CTAB method. The PCR reaction system was 20 μL in volume, containing approximately 10–100 ng of DNA template, 10 μL of 2× PCR mixing buffer, 1 μL each of forward and reverse primers (10 μM), and ddH_2_O up to 20 μL. The PCR reaction procedure was as follows: 95 °C denaturation for 3 min, 94 °C denaturation for 30 s, 58 °C annealing for 30 s, 72 °C extension for 20 s, and 75 °C extension for 5 min, for 28 cycles. PCR amplification was performed using a Bio-rad thermal cycler.

After the PCR reaction was completed, the PCR products were subjected to gel electrophoresis using an 8% polyacrylamide gel. The polyacrylamide gel consisted of 30% polyacrylamide solution, 10% ammonium persulfate (APS) solution, tetramethylethylenediamine solution (TEMED), 10× TBE solution, and ddH_2_O. After electrophoresis, the gel was first fixed with a fixative solution prepared with anhydrous ethanol and glacial acetic acid, then stained with 0.1% AgNO_3_ solution, and finally developed with an aqueous solution of sodium hydroxide, sodium tetraborate, and formaldehyde. The electrophoretic bands were counted after the gel was irradiated.

### 4.5. Data Analysis

The marker polymorphism information content (PIC) was calculated using POWERMARKER V3.25 software [31]. Based on the genotyping results, genetic distance was calculated using MEGA 7.0 software, and cluster analysis was performed using the UPGMA algorithm in MEGA software [32]. DNA fingerprints were then constructed. The population genetic structure of tomato germplasms were analyzed using STRUCTURE 2.3.4 software [33]. The parameters were set as follows: K value was set to 1–10, and each K value was calculated 10 times. The results were imported into Structure Selector (V2.3.4) software [34]. The optimal number of groups K was determined based on lnP(K) and ΔK value. Principal component analysis of the tomato population was performed using Plink 1.9 software [35].

## Figures and Tables

**Table 1:** InDel loci in the whole tomato genome.

Chromosome	Insertion	Deletion	Total	InDel/%
Chr01	7498	13,648	21,146	7.40
Chr02	8052	13,766	21,818	7.63
Chr03	12,137	14,721	26,858	9.40
Chr04	11,579	15,166	26,745	9.36
Chr05	11,378	14,644	26,022	9.11
Chr06	7865	12,458	20,323	7.11
Chr07	11,313	14,316	25,629	8.97
Chr08	10,758	14,980	25,738	9.01
Chr09	12,544	14,534	27,078	9.47
Chr10	10,925	13,165	24,090	8.43
Chr11	8542	11,011	19,553	6.84
Chr12	9342	11,454	20,796	7.28
Total	121,933	163,863	285,796	100.00

**Table 2:** Allele number and polymorphism information content (PIC) of 63 core InDel markers across 52 tomato accessions.

Chromosome	Marker	Allele Number	PIC	Chromosome	Marker	Allele Number	PIC
Ch01	T1M0546	3	0.390	Ch06	T6M3582	2	0.309
Ch01	T1M3899	2	0.355	Ch06	T6M3953	2	0.232
Ch01	T1M4575	2	0.298	Ch06	T6M4626	2	0.383
Ch01	T1M5533	3	0.402	Ch07	T7M0019	2	0.038
Ch01	T1M6009	2	0.307	Ch07	T7M0756	2	0.201
Ch01	T1M6565	2	0.258	Ch07	T7M1005	2	0.111
Ch02	T2M0112	2	0.134	Ch07	T7M5397	2	0.185
Ch02	T2M1114	2	0.074	Ch08	T8M2758	2	0.143
Ch02	T2M3180	2	0.124	Ch08	T8M3706	2	0.186
Ch02	T2M3592	2	0.373	Ch08	T8M5194	2	0.141
Ch02	T2M4420	2	0.145	Ch09	T9M0714	2	0.299
Ch02	T2M5113	2	0.284	Ch09	T9M0982	2	0.138
Ch03	T3M0497	2	0.378	Ch09	T9M1163	2	0.368
Ch03	T3M5319	2	0.359	Ch09	T9M2159	2	0.299
Ch03	T3M5408	3	0.376	Ch09	T9M3154	2	0.299
Ch04	T4M0210	2	0.368	Ch09	T9M3515	2	0.304
Ch04	T4M2158	2	0.384	Ch09	T9M3926	2	0.299
Ch04	T4M4727	2	0.384	Ch09	T9M4118	2	0.299
Ch04	T4M6000	2	0.384	Ch09	T9M4314	2	0.299
Ch05	T5M0251	2	0.299	Ch09	T9M4940	2	0.299
Ch05	T5M0701	2	0.285	Ch09	T9M5137	2	0.309
Ch05	T5M1237	2	0.357	Ch10	TaM0641	2	0.375
Ch05	T5M1244	2	0.358	Ch10	TaM6127	2	0.382
Ch05	T5M1709	2	0.291	Ch10	TaM6451	2	0.286
Ch05	T5M2207	2	0.356	Ch11	TbM0488	2	0.128
Ch05	T5M3207	2	0.090	Ch11	TbM0724	2	0.368
Ch05	T5M3712	2	0.280	Ch11	TbM1162	2	0.357
Ch05	T5M4208	2	0.344	Ch11	TbM2541	2	0.299
Ch05	T5M4704	2	0.356	Ch11	TbM3021	2	0.376
Ch06	T6M1523	2	0.295	Ch12	TcM0679	2	0.306
Ch06	T6M3024	2	0.194	Ch12	TcM6300	2	0.172
Ch06	T6M3003	2	0.193				

**Table 3:** DNA fingerprints of 52 tomato germplasm accessions based on 24 core InDel markers.

Accession	DNA Fingerprinting	Accession	DNA Fingerprinting
T1	111111111111311111111111	T27	212231221111221111111211
T2	111131111111231111111211	T28	212231221112221111211211
T3	111131011111231111211011	T29	422231222202221111210121
T4	212212122212221111112121	T30	012231211112231111112211
T5	212232122212221111220331	T31	210231221111221111111211
T6	210232122212221111122122	T32	222221221112231121211211
T7	222231122212221111212121	T33	422221211102231110222121
T8	412232022212221111102122	T34	422221211112231111212311
T9	410232122212221111102122	T35	212231211112221120112211
T10	212231121112221111210211	T36	422231111111221121202121
T11	212232122222221120111121	T37	212232122212221111210001
T12	212231121112231210121122	T38	212231122222221121022121
T13	212232121112221211212212	T39	212231121112221111211212
T14	212232121111221111210211	T40	222221121122221111211122
T15	212232122202221111112122	T41	010231221112221111211011
T16	212231121122201122022121	T42	422201211112231111212121
T17	212221121112222121112122	T44	212231221112221111121121
T18	220032122212221111111121	T43	212232222212221211211120
T19	212232121122231211111212	T45	012222222202222112211212
T20	210232122212221121121121	T46	212221222212221111212121
T21	210231220111221111111121	T47	210222221322222111221121
T22	212231221111221111111211	T48	212022022212221121201121
T23	322221211111222112112211	T49	210032022212221121211331
T24	212221221111221111111121	T50	212020022212221111111122
T25	212231221111221111111121	T51	212222020102221121121212
T26	210222211112230110212211	T52	212230021112221111211122

## Data Availability

All data are presented within the article.

## Supplementary Materials

The following supporting information can be downloaded at: https://www.mdpi.com/article/10.3390/plants15071118/s1, Figure S1: PCR amplification profiles of 52 tomato accessions using ten InDel primer pairs; Table S1: Primer information for total 255 developed Indel markers in Tomato. Table S2: Primer information for 63 core Indel markers in Tomato. Table S3: The main morphological characteristics and agronomic traits of 52 tomato varieties.

## Abbreviations

The following abbreviations are used in this manuscript:

InDel	Insertion-Deletion
PIC	Polymorphism Information Content
RFLP	Restriction Fragment Length Polymorphisms
RAPD	Amplified Polymorphic DNA
SSR	Simple Sequence Repeat
ISSR	Inter-Simple Sequence Repeat
SRAP	Sequence-Related Amplified Polymorphism
AFLP	Amplified Fragment Length Polymorphism
CAPS	Cleaved Amplified Polymorphic Sequences
SNP	Single-nucleotide polymorphism
