# Supplementary material for: Genome-Wide InDel Marker Development and Genetic Diversity Analysis of 52 Tomato Germplasm Accessions
Source: Plants (Basel). 2026 Apr 6;15(7):1118. doi: 10.3390/plants15071118 (PMC13074326; doi:10.3390/plants15071118)
Supplement: Supplementary file 1 [file plants-15-01118-s001.zip › Table S2.pdf]

Table S2. Primer information for 63 core Indel markers in Tomato.

chromosome	Marker	Forward primer (5′ - 3′)	Reverse primer (5′ - 3′)
Ch01	T1M0546	ACTCCACTAATGCAGACACTA	ATGTGGGACCCATTATCTAC
Ch01	T1M3899	CACCCCCACACTTTTATTTTCGTAA	GGTACCCCAATAGAAACGTTTTAAA
Ch01	T1M4575	TCGAACCCTGCAATCATCGC	ATGGTGAGGCTGCTTATTTG
Ch01	T1M5533	AGATGGGGAAAGGTTTCATC	GTGCCAAACACCATATCATC
Ch01	T1M6009	ACATGATTGCGGATGAGAAG	CCCCCTTCGTTACAGTAGATC
Ch01	T1M6565	CCTTCATTCACGTCAGGGAA	ATCGGGTTGCTCTAAGTTTG
Ch02	T2M0112	GGTTCTTGCGTGTCTTAGCC	CCTTGGATATTCTTACCCTG
Ch02	T2M1114	TGCTTGGTCTGTTTGTCTCT	AGAGTGCACCATTAGGAAAG
Ch02	T2M3180	GATCCACATCAAATTCAAAT	AGATAGCAGTTTCTACTTTC
Ch02	T2M3592	GATAAGGAATAGGGCAGAGAAT	GCCTATCGATACCACCACTGT
Ch02	T2M4420	CTCGATGCAACCAATGCTGAT	CCATTGTTCCATGCAGAAG
Ch02	T2M5113	TTCATGTTTAAAGCCCCGTT	CTGATGGAAACGTTGTATCTA
Ch03	T3M0497	TTACCGCAGCTACTACACTC	GTACTGAAAGAGCTGTGGCC
Ch03	T3M5319	GCAGTTTATATCATGTGCAA	AGTTGGGTTCGTTATTGCAT
Ch03	T3M5408	TTTGCTGCTATACTGCTGACA	TTCTTCCCTTCCATCAGTTCT
Ch04	T4M0210	AGGTACGTTTCTGCCGCGTC	ATAGTATCTCACAGGGAGAC
Ch04	T4M2158	CATACATATCACTTTCCTCATACTTTG	GCACTAAGTGTTTATTAAGCTCATG
Ch04	T4M4727	ACCAACACCACTCCTCTAGT	TCTTGCTTCATTTTGAGTGT
Ch04	T4M6000	ACTTAATTAGCATCAAAGCT	TGCTTTGAAGAATGCTAACT
Ch05	T5M0251	GTCATATTGGGAGTGATTTG	GTGTCTTGATTTAACTGAGG
Ch05	T5M0701	CCTGTATACAACGAACAATT	CCTTGGAAATGAGGTAATCG
Ch05	T5M1237	GATGAGGATGAATCTGAAGT	TACCTCCAATGATACTTTCG
Ch05	T5M1244	TTGATAACACATGTCACATGATTGG	TGGCAAGCTAGCTGGGATAG
Ch05	T5M1709	GTGTATTAGCAAATGTTACAG	TAGTTAGAAGGATGCATGAG
Ch05	T5M2207	GACGGTAGCTATTAAAGCGA	TGGATGAGAGTTGCAGAATT
Ch06	T6M3003	CCCAACCACCAATACTTTCC	AGAGGGTACTAAGAATTACC
Ch05	T5M3207	TCATATCTTGAACTACACCT	CCATGAGGAACTATGAGTGT
Ch05	T5M3712	TCACATGATACATGATTTAT	GCCCAATGTATCAACTCCAT
Ch06	T6M3953	AATTTCCCCAATCAAATAATCTTCAATTT	GGCAGCTTACGACTGGATAATCTAA
Ch05	T5M4208	GAATTCATGCTAGGACTTGG	GATTTAAAGTTCTTGGCAACT
Ch05	T5M4704	ATTCGCGAAGAGGTAGAGAG	TGGAAGGAGAATACATTGCG
Ch06	T6M1523	GCTGAAGTATGGTTGTCAGA	ATCATAAGTTGTTGATGTGC
Ch06	T6M3024	CAAGTGTTGTCTCCCACAAG	TTACATGGGGAACAAGGGTA
Ch06	T6M3582	GTAAAATCGTTGCCTTGGCA	ATTAAGAAAATCGGGAGCGA
Ch06	T6M4626	TGTCTAGTGCGGTTACCTCT	TATGCGCACAGGGTAGAACC
Ch07	T7M0019	AGAGCACAAAACCTTCTGAG	TTGAACTCTGCAGGAATGAG
Ch07	T7M0756	GGTGGAATAAGTGGTAGAGCT	GCTAACTTGAAATATTGCGTGTGATGC
Ch07	T7M1005	GTTAGTGGCTTACGTATCGTAATC	GGTTACAACTTACAAGAGCTGCAG
Ch07	T7M5397	ATCAACCGCAGAACAACATT	ACAAATTGCTCAAAAGCGTA

Ch08	T8M2758	GTTGTGTGGGATGACCAGAT	GAATCCTTCGTTCAATTGGT
Ch08	T8M3706	TTCTTGGTTGGGGCTTGGCG	CCCTGGCGGACAGAGTTGGT
Ch08	T8M5194	TCAAAATGCAGGGTGAAAGG	TTCATTCCGTAAGTGTCAAT
Ch09	T9M0714	GCTGAAAGGCCATTTCGTGT	CCTTCCTGGTTCCAAACAAT
Ch09	T9M0982	CCACTTCCTTTGGGGCTCGC	GTACAGACACTTAGGCACAA
Ch09	T9M1163	GGATCTTGTTTGGTTAATTGG	GGTTAAGAGACTAGGTCAAG
Ch09	T9M2159	GCACATAATCTCCCCCCTTGGA	CACTTATAATGGGCTGTAGT
Ch09	T9M3154	TGATATCGACAGGTTGGAGC	CTCGCACAACCCTAACAATA
Ch09	T9M3515	CCTTCTCCCCATAATACACA	GACCTCTAAGTTGAGGAACT
Ch09	T9M3926	CCGACGATAGACAATTAGACT	GCCCCCTTAATGGACCGTTG
Ch09	T9M4118	CGAATGCCTAATATGAACGTT	ATTGAGGAGGCATAGACATGG
Ch09	T9M4314	GGTCATAACCTTACCTTAAA	GTGTATTAACAATCGCCAAT
Ch09	T9M4940	GCAGACATGGACGAGTTGCC	CACTACCACATTAATGTTCC
Ch09	T9M5137	CCACATAAGAAGGGCCAATAA	CCACCACCATTAGCAATTGA
Ch10	TaM0641	GGTAGTGAGACTTGTTTTGA	AGTTTTTGAGTTGCATGTGT
Ch10	TaM6127	GAGTATGGCATCATCATAGCAC	CCGATTAATTTTATAACCAAATTG
Ch10	TaM6451	ACGGGCCATACCTATCTAGT	CCTAACATGCCATGATGACC
Ch11	TbM0488	TGAAAATATCTTTAAAGTTCGTGTGCA	CCTGCCCCAGGTGATTCATT
Ch11	TbM0724	CTGTAATCGTGTCTAATAGC	TTTGACCCTTCAAGTGGGGA
Ch11	TbM1162	CCCAACTTCAGAAATGCATA	ATGGTCGTAACTCCTAGATA
Ch11	TbM2541	TGCACGACGATCTGATACGG	GTAAGAGGCTGCTTCCACCC
Ch11	TbM3021	GGATAGTTACGAGTTACGAC	AACCCTTCGGAAATGTCACA
Ch12	TcM0679	GAGGACGACAACAACAACGA	GACATGCCACTTAGATCCACAA
Ch12	TcM6300	CTCAAGCAAATTGCAAGGTTGG	GGTCTTTAACAAGTTGTTGTATG
