# Supplementary figures and images for: Genome-Wide InDel Marker Development and Genetic Diversity Analysis of 52 Tomato Germplasm Accessions
Source: Plants (Basel). 2026 Apr 6;15(7):1118. doi: 10.3390/plants15071118 (PMC13074326; doi:10.3390/plants15071118)

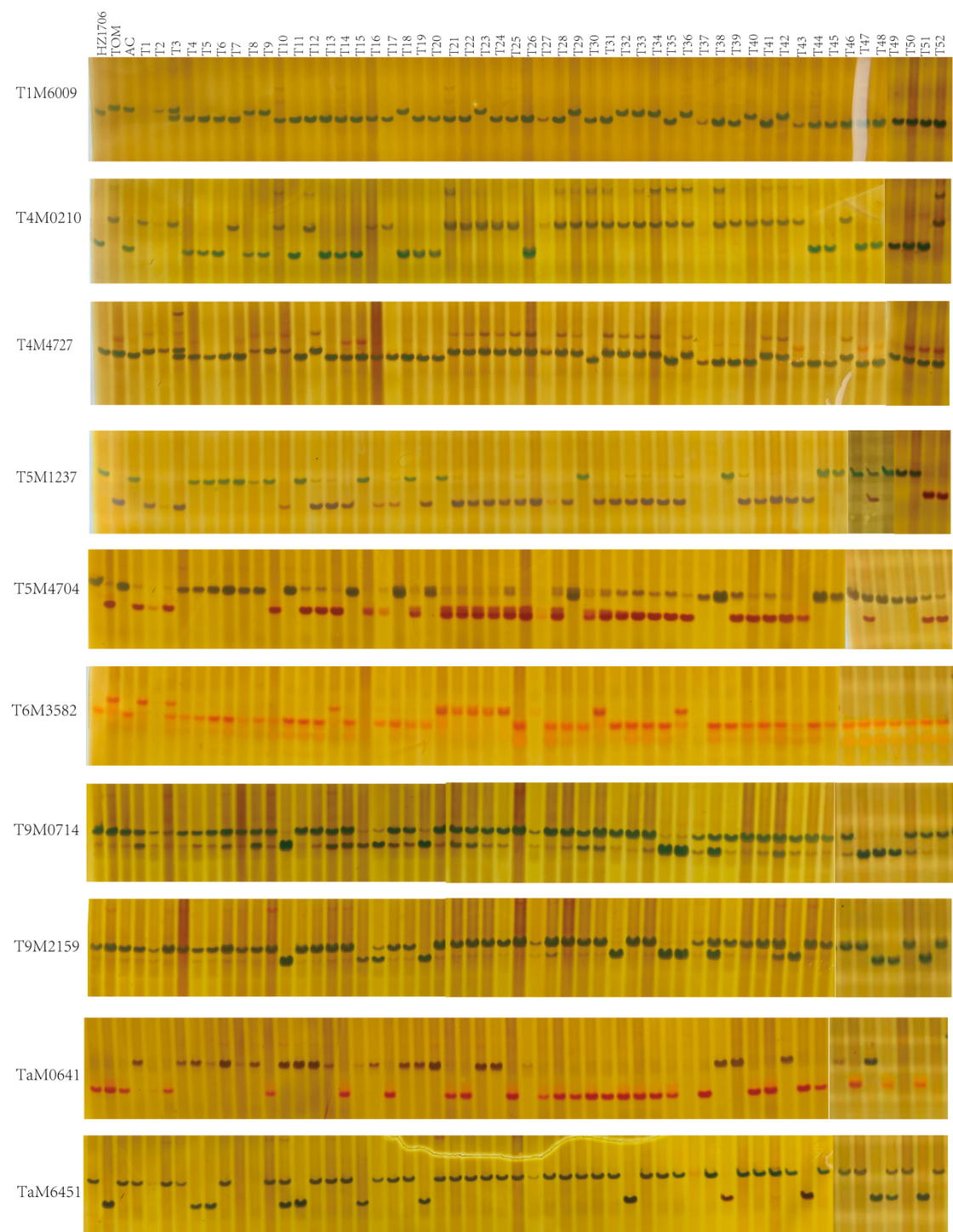

Figure S1 PCR amplification profiles of 52 tomato accessions using ten InDel primer pairs

Supplement: Supplementary file 1 [file plants-15-01118-s001.zip › Figure S1.pdf]
